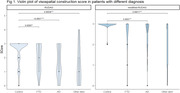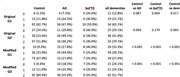# The modified Rowland Universal Dementia Assessment Scale for dementia screening in Peruvian population

**DOI:** 10.1002/alz.090904

**Published:** 2025-01-03

**Authors:** Nilton Custodio, Rosa Montesinos, Marco Malaga, Belen Custodio, Diego Chambergo‐Michilot, Diego Bustamante‐Paytan, Zadith Yauri, Katherine Aguero, Graciet Verastegui, Serggio Lanata, Christopher Butler, Giuseppe Tosto

**Affiliations:** ^1^ Unidad de Investigación, Instituto Peruano de Neurociencias, Lima, Perú, Lima, Lima Peru; ^2^ Escuela Profesional de Medicina Humana, Universidad Privada San Juan Bautista, Lima, LIMA Peru; ^3^ Unidad de diagnóstico de deterioro cognitivo y prevención de demencia, Instituto Peruano de Neurociencias, Lima, Perú, Lima, Lima Peru; ^4^ Instituto Peruano de Neurociencias, Lima, Lima Peru; ^5^ Cognitive Impairment Diagnosis and Dementia Prevention Unit, Peruvian Institute of Neurosciences, Lima, Lima Peru; ^6^ Cognitive Impairment Diagnosis and Dementia Prevention Unit, Instituto Peruano de Neurociencias, Lima, Perú, Lima, Lima Peru; ^7^ Facultad de Ciencias de la Salud, Universidad Científica del Sur, Lima Peru; ^8^ Memory and Aging Center, UCSF Weill Institute for Neurosciences, University of California, San Francisco, San Francisco, CA USA; ^9^ Global Brain Health Institute, San Francisco, CA USA; ^10^ Imperial College London, London United Kingdom; ^11^ The George Institute for Global Health, Sydney Australia; ^12^ Gertrude H. Sergievsky Center, Taub Institute for Research on the Aging Brain, Departments of Neurology, Psychiatry, and Epidemiology, College of Physicians and Surgeons, Columbia University, New York, NY USA; ^13^ Taub Institute for Research on Alzheimer’s Disease and the Aging Brain, Vagelos College of Physicians and Surgeons, Columbia University, New York, NY USA

## Abstract

**Background:**

Transcultural adaptations of brief cognitive screening tools (BCTs) involve the development of alternate versions that are psychometrically equivalent to the original, while being linguistically and culturally adapted to a new sociodemographic context. The RUDAS (Rowland Universal Dementia Assessment Scale) is less affected by culture, language, and education compared with other BCTs. However, several studies have reported an effect of education on RUDAS scores. In Peru, performance on visuospatial construction and judgement items of the RUDAS may be particularly low amongst rural communities. The main aim of this study was to evaluate the performance of a culturally‐refined version of the Peruvian version of RUDAS (RUDAS‐PE), the RUDAS‐PEm. We tested how well the RUDAS‐PEm distinguished between controls and patients with Alzheimer’s disease (AD), behavioral variant frontotemporal dementia (bvFTD), and other types of dementia, compared to the RUDAS‐PE.

**Method:**

To create the RUDAS‐PEm, we modified two domains of RUDAS‐PE, visuo‐spatial construction and judgement, to psychometrically‐congruent alternatives that are more appropriate for the Peruvian context. We changed the ‘cube drawing’ task (visuospatial construction) to drawing a circle, rhombus, and rectangle. The ‘crossing the street in transit’ task (judgement) was changed to one asking patients to tell the difference between sugar and vinegar and to explain how to locate a new friend in a new city.

**Results:**

We included 110 controls and 87 patients with dementia: 54 with AD, 20 with bvFTD, and 13 with other dementias. Mean age was 70 (SD 7.8) and mean education was 14.2 years (SD 4.1). For visuospatial construction in RUDAS‐PE, there were significant differences between the scores of controls and AD patients (<0.001), bvFTD patients (0.02), and other dementias (p<0.001) (figure 1). For RUDAS‐PEm, no significant differences were found between scores of controls and bvFTD patients. For the judgment item both versions had good discrimination, but RUDAS‐PEm showed better performance (table 1). The times to complete both versions and the AUC for diagnosis of AD versus controls, bvFTD versus controls, and all dementias versus controls, were similar in both versions.

**Conclusion:**

The RUDAS‐PEm including modification of the judgment item shows modest improvement in detecting dementia.